# Sarcopenia status as assessed by hand grip strength enhances prediction of post-operative outcomes in hip fracture patients

**DOI:** 10.1007/s40520-025-03262-7

**Published:** 2025-12-29

**Authors:** James Prowse, Sharlene Jaiswal, Andrew Chaplin, Sarah Langford, Avan A. Sayer, Nicola Sinclair, Mike R. Reed, Miles D. Witham, Antony K. Sorial

**Affiliations:** 1 AGE Research Group, Faculty of Medical Sciences, Translational and Clinical Research Institute, Newcastle University, Newcastle Upon Tyne, UK; 2 NIHR Newcastle Biomedical Research Centre, Newcastle Upon Tyne NHS Foundation Trust, Cumbria Northumberland Tyne and Wear NHS Foundation Trust and Newcastle University, Newcastle Upon Tyne, UK; 3 Department of Trauma and Orthopaedics, Northumbria Healthcare NHS Foundation Trust, Newcastle Upon Tyne, UK; 4 Institute for Cell and Molecular Biosciences, International Centre for Life, Newcastle University, Newcastle Upon Tyne, NE1 3BZ UK

**Keywords:** Sarcopenia, Hand grip strength, Hip fracture, Post-operative outcomes, Prognostication

## Abstract

**Background:**

Measuring hand grip strength (HGS) at admission can indicate probable sarcopenia in acute hip fracture and may help predict adverse post-operative outcomes.

**Aims:**

To assess whether HGS is independently associated with adverse post-operative outcomes and enhances risk prediction when combined with the Nottingham Hip Fracture Score (NHFS).

**Methods:**

Routinely collected National Hip Fracture Database data were combined with HGS at a single, high-volume orthopaedic trauma centre. Sarcopenia status was categorised per 2019 European Working Group thresholds as non-sarcopenic, probable sarcopenia, or unable to complete assessment. Binary logistic regression models and receiver-operating-characteristic (ROC) curves assessed prognostic value of HGS assessment.

**Results:**

We analysed data from 282 patients, mean age 83.2 ± 9.2 years; 200 (71%) were women. In univariate analysis, probable sarcopenia (low HGS) was associated with 120-day mortality, 30- and 120-day residential status, prolonged length of stay and post-operative delirium. In multivariate analysis including the NHFS, probable sarcopenia was associated with 120-day mortality, 120-day residential status and post-operative delirium. Combining NHFS and HGS assessment improved discrimination for 120-day mortality (c-statistic 0.79 [95%CI 0.73–0.86]), 30-day residential status (0.85 [95%CI 0.80–0.90]) 120-day residential status (0.89 [95% CI 0.85–0.94]) and post-operative delirium (0.91 [95%CI 0.87–0.94]). Inability to complete HGS assessment was associated with worse prognostic outcomes than low HGS.

**Discussion:**

Sarcopenia is a useful additional predictor of post-operative outcomes in hip fracture, especially post-operative delirium. Inability to complete HGS assessment may indicate even higher risk.

**Conclusion:**

HGS is an inexpensive, feasible and quick adjunct to the NHFS. Further research is required to validate a combined NHFS-HGS score.

**Supplementary Information:**

The online version contains supplementary material available at 10.1007/s40520-025-03262-7.

## Introduction

Hip fracture is one of the most common traumatic injuries in the UK, with substantial implications for patients, including functional impairment, increased care requirements and a high mortality risk. Total annual numbers are predicted to rise, increasing disease burden and costs to the National Health Service (NHS) [1].

In the UK, standardisation of hip fracture patient care through the National Hip Fracture Database (NHFD) has improved patient outcomes [2]. However, adverse outcomes including death, prolonged length of stay (LOS) and post-operative complications such as delirium remain common and clinically important. Improved prediction of adverse outcomes can identify at-risk patients, enabling targeted intervention and improved clinician case mix.

The Nottingham Hip Fracture Score (NHFS) is widely used to estimate the risk of 30-day post-operative mortality at admission [3]. Independent validation has shown the NHFS performs moderately well in predicting mortality, consistently well in predicting functional outcomes but less well in predicting LOS and post-operative complications [4]. The NHFS does not consider muscle strength, however it has been reported that sarcopenia (the age-related loss of muscle mass and strength) is a key driver of falls and a component of physical frailty [5]. The prevalence of sarcopenia in older hip fracture patients varies between 17–46% depending on the population and definition [6–8].

While there is no universally agreed definition of sarcopenia, the revised European Working Group on Sarcopenia in Older People (EWGSOP2) definition recommends assessment of muscle strength to identify probable sarcopenia. Diagnosis can then optionally be confirmed by assessment of low muscle quantity or quality, which can be expensive, time consuming and logistically difficult [9]. In a clinical setting, our recent systematic review suggests muscle mass assessment offers no additional prognostic information to muscle strength in hip fracture[10]. Muscle strength assessment most commonly involves the use of a hand-held dynamometer to measure hand grip strength (HGS) or measurement of sit to stand time. In patients with hip fracture, sit to stand time is not feasible. However, available literature suggests HGS is strongly correlated with lower extremity muscle power and calf cross-sectional area and is therefore an important proxy for skeletal muscle strength in patients with hip fracture[11].

For HGS to be of utility in clinical practice, it must be feasible to measure in a broad range of patients with hip fracture and associated with a range of adverse outcomes. Most importantly it should add prognostic value to existing prognostication tools, or it should trigger a change in management through interpretation of the measurement. As we have already demonstrated feasibility of measuring HGS in the clinical setting, this study evaluated the association between probable sarcopenia as assessed by HGS and post-operative hip fracture outcomes, and furthermore, whether sarcopenia status as assessed by HGS improved the discriminant ability of the NHFS [10].

## Patients and methods

### Study population

NHFD data was collected prospectively for hip fracture patients aged ≥ 60 years admitted to Northumbria Healthcare NHS Foundation Trust between 1st March 2020 to 30th March 2022. Hip fracture was defined as primary proximal femoral fracture, excluding other fracture types. Patients were excluded if HGS was not measured and there was no clinical reason for not attempting assessment. Exclusion criteria included organisational or logistic factors (staffing shortages, patient location, language barriers and refusal).

### Sarcopenia assessment

HGS was routinely assessed pre-operatively by specialist physiotherapists following a standardised protocol using a Jamar hydraulic dynamometer (Sammons Preston, Bolingbrook, IL, USA) [10]. The maximum grip strength from two attempts in each hand was recorded. HGS was analysed both as a continuous variable and as a categorical variable by classification of patients by probable sarcopenia status using the EWGSOP2 criteria (≥ 27 kg men, ≥ 16 kg women) with a further category for inability to complete HGS assessment.

### Patient baseline characteristics and post-operative data

Clinical staff calculated Abbreviated Mental Test Score (AMTS) for cognitive impairment that could indicate either acute delirium or pre-existing dementia, Rockwood Clinical Frailty Score (CFS) and 30-day mortality risk as assessed by NHFS at admission [3, 12, 13]. ASA grade was evaluated by anaesthetic teams pre-operatively. Baseline patient characteristics including age, sex, type of fracture, pre-fracture residential status, and mobility were obtained from clinical records.

### Post-operative outcome data

We assessed mortality, post-operative residential status, post-operative mobility, LOS and delirium. Post-discharge outcomes at 30 and 120 days, including mortality, residential status and mobility were collected from telephone interviews and clinical records. Residential status was classified as own home/sheltered housing post-operatively or other residence/deceased. Pre-fracture and 120-day mobility was classified into five categories describing level of mobility in descending order of function. Outcomes other than mobile outdoor without aids were deemed as reduced mobility. LOS was categorised as ‘short’ or ‘prolonged’ using cohort median LOS for episode of care as cut-off. Potential postoperative delirium was assessed within 72 h following surgery using the 4AT rapid clinical test [14].

### Ethical approval

This project was deemed to not require ethics committee review as analyses did not require any new patient contact and utilised routinely collected audit data [15]. All data were managed in accordance with Caldicott Principles and approval was provided from the Caldicott guardian at Northumbria Healthcare NHS Foundation Trust (local administrative reference C3667).

## Statistical analysis

### Descriptive and univariate analyses

Statistical analyses were conducted using IBM Statistical Package for the Social Sciences (SPSS) version 27 (IBM Corp, Armonk, USA) and Prism v9.0 (GraphPad Software, California, USA). Parametric data are displayed as mean ± standard deviation, with non-parametric data displayed as median (interquartile range, IQR). Normality of continuous variables was assessed by visual inspection of histograms and Q-Q plots. Chi-square testing was used to compare categorical variables. *P* values < 0.05 were considered statistically significant. For continuous variables, Pearson’s or Spearman’s rank correlation testing was used to assess parametric or non-parametric data respectively. Associations between binary and continuous variables were assessed using t-testing for parametric data and Mann–Whitney-U testing for non-parametric data. To ensure the dataset analysed was representative of the overall cohort univariate analyses were performed comparing included and excluded cohorts. We also assessed for significant differences between sexes and for any identified significant differences, separate analyses were performed.

### Outcome prediction analysis

Binary logistic regression analysis assessed whether probable sarcopenia was a predictor for adverse post-operative outcomes, both independently and when combined with NHFS. Post-operative outcomes were examined in separate binary logistic regression models. Variables were dichotomised, with the optimal outcome identified as reference. Since NHFS incorporates pre-fracture AMTS, residence, age and sex, we did not include these variables again as independent predictors. Results were reported as odds ratio [95% confidence intervals].

Correlation coefficients between independent variables were evaluated to mitigate multicollinearity, and scatter plots and error bars illustrating the relationship between independent and dependent variables. Only patients with complete data were included in regression analyses. Receiver operating characteristic (ROC) curves and C-statistics with 95% confidence intervals compared the discriminative ability of NHFS alone and NHFS combined with probable sarcopenia. We defined C-statistics as > 0.80 indicating a good predictive ability, 0.60 > X ≤ 0.80 moderate and < 0.60 poor.

## Results

### Study participants

Between 1st March 2020 and 30th March 2022, the National Hip Fracture Database recorded 1569 patients with acute hip fracture requiring surgery at our centre. We excluded 1278 patients with absent grip strength due to organisational factors (low clinical staffing n = 814, 63.7%, language barriers n = 20, 1.6% refusal n = 17, 1.3%) or lack of clinical reason for exclusion (n = 427, 33.4%), resulting in a cohort of 282 patients with surgically managed primary proximal femoral fractures. Patients were classified into: probable sarcopenia (n = 99), non-sarcopenic (n = 74) and unable to complete HGS assessment (n = 109). A flow chart of study subjects is shown in Supplementary Fig. 1, with details regarding fracture and operation types for our population and excluded cohorts in Supplementary Table 1.

**Fig. 1:**
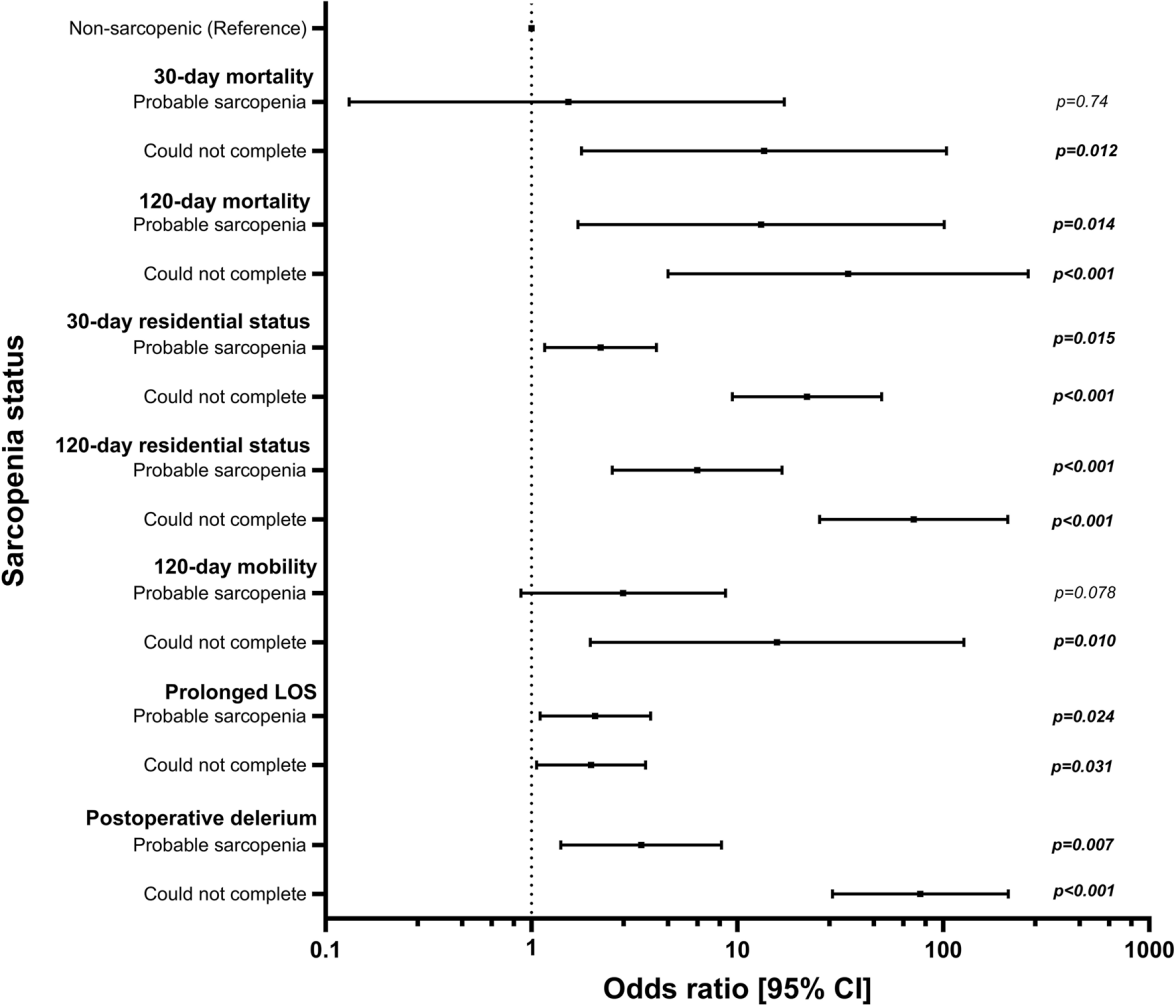
Box and whisker plot showing binary logistic regression models summarising association between sarcopenia status and selected post-operative outcomes. LOS:Length of stay (defined as > 15 days), delirium (Determined using 4AT score of ≥ 4). Probable sarcopenia assessed by hand grip strength ≥ 27 kg in men, or ≥ 16 kg in women

### Baseline characteristics

The mean age of our cohort was 83.2 ± 9.2 years. There were 200 women (70.9%), and 82 men (29.1%). Within this cohort, 171 patients (60.6%) had intracapsular fractures and 111 patients (39.4%) had extracapsular fractures. The majority (56.0%) of our patients had some indoor mobility but never mobilised outside without aids. Pre-operatively, median AMTS was 7.0 (2.0–10.0), mean CFS score was 4.4 ± 1.4, with 135 patients (49.4%) considered frail (CFS score ≥ 5). The mean NHFS score was 5.3 ± 1.7, with 228 patients (59.2%) graded ASA 3 (severe systemic disease) on pre-operative assessment. (54.6%). Table 1 summarises our cohort baseline characteristics.

**Table 1:** Cohort baseline characteristics

Variable		Baseline Characteristic (n = 282)
Age, years		83.2 ± 9.2
Sex, n women (%)		200 (70.9)
Pre-fracture residential status, n (%)	Own home/sheltered housing	203 (72.0)
	Residential care home	75 (26.6)
	Nursing care home	4 (1.4)
Pre-fracture mobility, n (%)	Mobile outdoor without aids	67 (23.8)
	Mobile outdoor with aids	52 (18.4)
	Some indoor mobility	158 (56.0)
	No mobility	5 (1.8)
Pre-operative AMTS (IQR)		7.0 (2.0–10.0)
CFS Score		4.4 ± 1.4
Classified as frail (CFS ≥ 5), n (%)		135 (49.4)
NHFS		5.3 ± 1.7
ASA Grade, n (%)	1	5 (1.8)
	2	49 (17.4)
	3	167 (59.2)
	4	61 (21.6)
Fracture type, n (%)	Intracapsular	171 (60.6)
	Extracapsular	111 (39.4)
Operation type, n (%)	Hemiarthroplasty	154 (54.6)
	Total hip replacement	12 (4.3)
	Internal fixation	114 (40.4)
	Other	2 (0.7)

Cohort baseline characteristics NHFS: Nottingham Hip Fracture Score, CFS: Clinical frailty score, AMTS: Abbreviated Mental Test Score, ASA: American Society of Anaesthesiologists

### Feasibility of sarcopenia assessment

For our cohort of 282 patients, sarcopenia status from HGS was assessed in 173 patients (61.3%). The most common reason for inability to complete assessment was ‘confusion, dementia or delirium’ (91.7%). We identified 99 patients (35.1%) with probable sarcopenia. A greater proportion of men (47.6%) than women (30.0%) had probable sarcopenia. There were significant differences between sexes in NHFS (*p* < 0.001), HGS (*p* < 0.001) and sarcopenia status (*p* = 0.018). Subsequent analysis for these variables was therefore split by sex.

### Post-operative outcomes

Within our cohort, only 3 patients with grip strength assessment died within 30-days, precluding multivariable and concordance statistical analysis of 30-day mortality. Other post-operative outcomes analysed are summarised in Table 2.

**Table 2:** Summary of cohort post-operative outcomes by sarcopenic status

Outcome assessed	Status	Pre-operatively	Non-sarcopenic	Probable sarcopenia	Unable to complete	p
30-day mortality (n = 282), n (%)	Alive	-	73 (98.6)	97 (98.0)	92 (84.4)	** < 0.001**
120-day mortality (n = 282), n (%)	Alive	-	73 (98.6)	84 (84.8)	74 (67.9)	** < 0.001**
30-day residential status (n = 282), n (%)	Own home/sheltered housing	203 (72.0)	49 (66.2)	47 (47.5)	9 (8.3)	** < 0.001**
	Other residence or death	79 (28.0)	25 (33.8)	52 (52.5)	100 (91.7)	
120-day residential status (n = 245), n (%)	Own home/sheltered housing	203 (72.0)	51 (89.5)	48 (57.1)	11 (10.6)	** < 0.001**
	Other residence or death	79 (72.0)	6 (10.5)	36 (42.9)	93 (89.4)	
120-day mobility (n = 186), n (%)	Mobile outdoor without aids	67 (23.8)	10 (18.9)	5 (7.7)	1 (1.5)	**0.003**
	Any other mobility	215 (76.2)	43 (81.1)	60 (92.3)	67 (98.5)	
LOS (n = 282), n (%)	Short (0–15 days)	-	48 (64.9)	47 (47.5)	53 (48.6)	**0.045**
	Prolonged (≥ 15 days)	-	26 (35.1)	52 (52.5)	56 (51.4)	
Median 4AT Score (IQR)		-	0.0 (0.0–0.25)	1.0 (0.0–4.0)	4.0 (4.0–4.0)	** < 0.001**
Post-operative 4AT Score (n = 282), n (%)	Delirium unlikely (0–4)	-	67 (90.5)	73 (73.7)	12 (11)	** < 0.001**
	Possible delirium (4–12)	-	7 (9.5)	26 (26.3)	97 (89)	

Summary of cohort post−operative outcomes by sarcopenic status. LOS: Length of stay 4AT: Abbreviated mental test score: presence of delirium considered likely if 4AT score ≥4/10. Probable sarcopenia assessed by hand grip strength ≥27kg in men, or ≥16kg in women

### Comparison of included and excluded cohorts

We compared included patients (n = 282) and excluded patients (n = 1287). Significant differences were seen between groups in pre- and post-operative delirium assessment scores, with included patients having a lower AMTS (p < 0.001) and lower 4AT score (p < 0.001). Differences were also seen in fracture type, surgical procedure, age and NHFS. Full comparisons are reported in supplementary Table 2.

### Continuous association between hand grip strength, baseline characteristics and postoperative outcomes

In women, increasing age was associated with lower maximum HGS (r = −0.50, *p* < 0.001). A higher maximum HGS was noted in women admitted with lower ASA score (*p* < 0.001) and from own home (p = 0.002). For both sexes, lower maximum HGS was associated with worse mobility (*p* < 0.001), lower AMTS (men r = 0.33, *p* = 0.011, women r = 0.33 *p* < 0.001), higher CFS (men r = −0.30, *p* = 0.026, women r = −0.51 *p* < 0.001). On visual inspection this trend in CFS was present only for men (*p* = 0.23). These analyses are detailed in supplementary Table 3 and 4. In continuous analysis for female patients there was an association between lower HGS and worse 4AT delirium assessment score (r = 0.33, p < 0.001), prolonged LOS (r = −0.33, p = 0.046), increased 120-day mortality (p = 0.007) and residential status at all timepoints (all p < 0.001). In male patients there was an association between lower HGS and worse 4AT delirium assessment score (r = −0.46, p < 0.001), as well as increased length of stay (r = −0.35, p = 0.008).

### Univariate associations between hand grip strength and post-operative outcomes

To facilitate inclusion of those unable to complete GS measurement, and to enable data from both sexes to be combined, outcomes were subsequently compared between sarcopenia categories. Probable sarcopenia as indicated by low HGS was associated with increased risk of 120-day mortality, adverse 30- and 120-day residential status, worse 120-day mobility, prolonged LOS and post-operative delirium. Inability to complete assessment was associated with the same outcome profile for 30-day mortality. Binary logistic regression models summarising the associations between probable sarcopenia, inability to complete HGS assessment and post-operative outcomes are shown in Fig. 1. All regression models were repeated with stratification by sex with similar results, detailed in supplementary tables 4 and 5.

### Additional discriminative ability of HGS when combined with NHFS

In multivariable analyses, the NHFS alone was significantly associated with all the outcomes assessed. These associations remained significant in the combined model. In this model probable sarcopenia (low HGS) was significantly associated with 120-day mortality, 120-day residential status and post-operative delirium. Inability to complete HGS assessment was significantly associated with 120-day mortality, 30- and 120-day residential status and post-operative delirium. Due to the small numbers of patients with recorded HGS who died within 30-days of admission (n = 3), multivariable analysis of 30-day mortality was not possible. Full analyses are presented in Table 3.

**Table 3:** Multivariable analysis comparing association of NHFS alone and NHFS with addition of probable sarcopenia (low hand grip strength) assessment status for post-operative outcomes

Outcome	Model 1: NHFS alone		Model 2: NHFS + Sarcopenia status					
			NHFS		Probable sarcopenia (Low HGS)		Unable to complete HGS assessment	
	OR [95% CI]	P value	OR [95% CI]	P value	OR [95% CI]	P value	OR [95% CI]	P value
Mortality at 30 d (n = 268)	2.56 [1.62–4.06]	** < 0.001**	2.12 [1.28–3.50]	**0.003**	0.77 [0.065–9.08]	0.84	3.85 [0.45–32.84]	0.22
Mortality at 120 d (n = 282)	2.13 [1.59–2.85]	** < 0.001**	1.80 [1.31–2.48]	** < 0.001**	8.07 [1.01–64.27]	**0.049**	11.17 [1.49–93.01]	**0.019**
30-day residential status (n = 268)	2.46 [1.93–3.12]	** < 0.001**	1.95 [1.52–2.51]	** < 0.001**	1.33 [0.65–2.75]	0.44	8.06 [3.06–21.18]	** < 0.001**
120-day residential status (n = 232)	2.71 [2.06–3.56]	** < 0.001**	1.90 [1.40–2.57]	** < 0.001**	6.80 [2.12–21.85]	**0.001**	37.45 [10.64–131.83]	** < 0.001**
120-day mobility (n = 186)	2.14 [1.51–3.03]	** < 0.001**	1.93 [1.32–2.83]	** < 0.001**	2.48 [0.71–8.71]	0.16	4.22 [0.43–41.01]	0.22
Prolonged LOS > 15 days (n = 282)	1.27 [1.09–1.48]	**0.003**	1.25 [1.04–1.49]	**0.016**	1.93 [0.99–3.77]	0.055	1.34 [0.64–2.81]	0.43
Post-operative delirium by 4AT (n = 282)	2.30 [1.82–2.90]	** < 0.001**	1.55 [1.18–2.04]	**0.002**	3.14 [1.10–8.98]	**0.033**	60.31 [18.84–193.06]	** < 0.001**

Multivariable analysis comparing association of NHFS alone and NHFS with addition of HGS assessment status for post-operative outcomes. HGS: Hand grip strength NHFS: Nottingham hip fracture score LOS: Length of Stay 4AT: Abbreviated mental test score. Presence of delirium considered likely if 4AT score ≥ 4/1. Probable sarcopenia assessed by hand grip strength ≥ 27 kg in men, or ≥ 16 kg in women.

In our concordance analyses, NHFS alone was a moderate predictor of 30- and 120-day mortality and a good predictor of adverse 120-day mobility status, 30- and 120-day residential status. Probable sarcopenia alone was a moderate predictor of 120-day mortality and 120-day mobility, and a good predictor of post-operative delirium, 30- and 120-day residential status. Combining the NHFS and sarcopenia status increased predictive ability for 120-day mortality, 30-day and 120-day residential status, prolonged LOS, and post-operative delirium. Prediction of prolonged LOS remained poor. Full results for concordance analyses are presented in Table 4. Supplementary Figs. 2–7 display the ROC curves.

**Table 4:** Summary of concordance analyses performed for post-operative outcomes

Outcome	Model used					
	NHFS alone		Probable sarcopenia alone		NHFS + probable sarcopenia	
	C Statistics [95% CI]	P- value	C Statistics [95% CI]	P- value	C Statistics [95% CI]	P- value
Mortality at 120 d (n = 282)	0.752 [0.680–0.824]	** < 0.001**	0.716 [0.647–0.786]	** < 0.001**	0.792 [0.728–0.855]	** < 0.001**
30-day residential status (n = 268)	0.808 [0.752–0.861]	** < 0.001**	0.778 [0.722–0.834]	** < 0.001**	0.850 [0.803–0.897]	** < 0.001**
120-day residential status (n = 232)	0.816 [0.760–0.871]	** < 0.001**	0.848 [0.797–0.899]	** < 0.001**	0.893 [0.851–0.936]	** < 0.001**
120-day mobility (n = 186)	0.881 [0.820–0.942]	** < 0.001**	0.747 [0.634–0.861]	** < 0.001**	0.872 [0.799–0.944]	** < 0.001**
Prolonged LOS > 15 days (n = 282)	0.581 [0.513–0.649]	**0.022**	0.578 [0.510–0.647]	**0.027**	0.598 [0.531–0.666]	**0.006**
Post-operative delirium by 4AT (n = 282)	0.782 [0.728–0.836]	** < 0.001**	0.878 [0.835–0.921]	** < 0.001**	0.905 [0.868–0.942]	** < 0.001**

C statistics as a marker of prognostic value for post-operative outcomes, recorded three models used in binary regression analysis. C statistics scored between 0.5–1.0, with a greater C statistic indicating greater prognostic value. HGS: Hand grip strength NHFS: Nottingham hip fracture score LOS: Length of stay 4AT: Abbreviated mental test score. Presence of delirium considered likely if 4AT score ≥ 4/10. Probable sarcopenia assessed by hand grip strength ≥ 27 kg in men, or ≥ 16 kg in women.

## Discussion

### Key findings

We present a unique dataset assessing HGS in acute hip fracture and associations with a broad range of post-operative outcomes. We employ this approach to enhance risk stratification using existing prognostic scores in routine clinical use. We have previously demonstrated that HGS assessment is feasible in older people with hip fracture, but challenges exist due to organisational factors and pre-operative delirium [10]. In our cohort, probable sarcopenia alone was a predictor for 120-day mortality, 30-day residential status, 120-day residential status, prolonged LOS and post-operative delirium in univariate analysis. When combined with the NHFS, sarcopenia status added prognostic value to the outcomes of 120-day mortality, 30-day/120-day residential status and post-operative delirium. The NHFS and HGS combined models significantly improved prediction of post-operative delirium compared to NHFS alone, but remained poor predictors of prolonged LOS. This may be due to the most frail, comorbid patients being discharged quickly due to limited benefit from further interventions, with our dichotomisation of LOS for analytical purposes reducing statistical power and concealing the bimodal distribution of LOS. Prediction of 120-day mobility did not improve in combined models, which may be reflective of our categorisation of mobility status, and an inherent limitation of the study. Notably, inability to complete grip strength assessment was also associated with these outcomes as well as worse 120-day mobility. This patient group was associated with increased frailty and possible cognitive impairment or delirium on admission. The association with post-operative delirium was greatly increased within this subgroup, and pre-operative cognitive impairment may have precluded HGS assessment.

### Comparable research

There is a paucity of studies investigating the association between patient sarcopenia status as assessed by HGS and post-operative outcomes in hip fracture. A 2019 review by Xu et al. identified four studies linking low HGS with adverse post-operative functional outcomes [16]. Menéndez-Colino et al. and Irisawa et al. reported an association between low HGS, mortality and motor functional independence score, consistent with our results [17, 18]. No studies have evaluated associations between sarcopenia and residence post-discharge. We postulate the baseline cognitive impairment in our cohort was a determiner of post-operative care needs. It is challenging to compare our assessment of mobility to existing literature. Prior studies have noted moderate correlations between HGS and 6-month Barthel Index scores [19].

In our study, increased LOS was correlated with lower HGS in both men and women (supplementary Table 4). This contrasts with results by Selakovic et al., who reported no association between sarcopenia status and LOS in their analyses [20]. It is possible that categorisation of sarcopenia status inhibited the power of Selakovic et al.’s study. However, our data also included time spent in post-acute hospital care (including rehabilitation hospitals considered part of the centre), limiting direct comparison. The median length of stay in our patients (15 days) was lower than the national average reported by the NHFD in this time period (19.7 days, February 2020) [21].

Our prevalence of post-operative delirium was higher than that reported in the NHFD (53.9% vs 30%) [21]. The association between sarcopenia and delirium may relate to similar risk profile for both conditions, including poor nutritional status, low functional mobility and pathophysiological similarities in inflammatory mediators and reactive oxygen species production [22].

The NHFS performed consistently well in prediction of mortality, residential status, mobility, and post-operative delirium across. Despite the strength of the NHFS in outcome prediction, C-statistics for outcomes predicted by HGS alone are comparable to those of the NHFS. The NHFS is widely used and extensively validated in predicting 30-day mortality and previous work from our group has demonstrated association with adverse post-operative outcomes [4, 23, 24]. The NHFS accounts for age, with sarcopenia also more prevalent in older people [9]. It is likely that independent association between sarcopenia and outcomes has been weakened by the collinearity between both independent variables. As indicated by previous work, it may be clinically beneficial to broaden the predictive scope of the NHFS [4].

### Strengths

Our study had several strengths. We incorporated HGS assessment following a robust training programme [10]. Assessment at admission was designed to provide an objective measure of grip strength as close to the injury as clinically feasible, thus minimising any impact of loss of muscle strength secondary to a catabolic patient state post-injury. Our HGS assessment was incorporated into routine care, allowing for identification of probable sarcopenia at an early timepoint where targeted intervention could be employed. We present a unique dataset, utilising a validated assessment of muscle strength routinely alongside NHFS.

Our assessment of a plethora of post-operative outcomes is novel compared to existing studies in this field. Furthermore, implementation of HGS testing facilitated identification of a distinct group of patients unable complete HGS assessment who are at even greater risk of adverse outcomes than those with probable sarcopenia and may warrant targeted intervention.

### Limitations

Our study also had limitations. The study was performed in a single centre and staffing variations, the COVID-19 pandemic and organisational factors limited collection of HGS measurements. This contributed to a smaller sample size, limiting our analyses and ability to stratify by sex. The use of some retrospective data collection led to some missing data from analyses, and inability to incorporate possible covariates such as height or body mass index (BMI).

There were statistically significant differences between our included and excluded cohorts in terms of baseline cognitive status, fracture type and surgical procedure. The documented reason for exclusion of the majority of patients was subsequent to inadequate clinical staffing, and we must take at face value this organisational reason for exclusion. Clinical inability to complete assessment was most commonly secondary to ‘confusion, dementia or delirium’. Our results show a greater association with adverse outcomes and hence omission from analyses may have introduced healthy user bias and underestimated the association between HGS and adverse outcomes. Our included cohort had a poorer baseline cognitive status (assessed using AMTS) and post-operative delirium score (assessed using 4AT) than our excluded cohort, indicating that presence of delirium was not the predominant reason for exclusion of these patients.

Our included cohort had a greater proportion of intracapsular fractures, than extracapsular fractures than the excluded cohort . This may have driven significant differences in the choice of surgical procedure. Furthermore, extracapsular fractures are recognised to be more common. Our study included predominantly elderly female patients, for whom osteoporosis is known to accelerate loss of trabecular bone and therefore increase rates of intracapsular fractures. While not captured, fractures within our population may have been subsequent to simple falls from standing height, a common cause of intracapsular fracture. Finally, high-energy or polytrauma patients may have been immediately transferred to a major trauma centre, and therefore would not be represented in our data, leading to potential exclusion bias in our results. Nevertheless, our results suggest that further work is worthwhile to refine implementation of handgrip strength testing in clinical services. Increasing the proportion of patients receiving this evaluation would in turn generate more complete data in different populations at different trauma centres, addressing any concerns around the generalisability of findings.

Inability to assess HGS in a proportion of our patients is recognised to be a limitation of this study. Alternative objective methods of muscle assessment such as assessing muscle mass via dual-energy x-ray absorptiometry or bioelectrical impedance analysis appear feasible in hip fracture, although current evidence suggests muscle mass assessment offers no additional prognostic information when compared to assessment of strength [25]. However, the application of such investigations in this cohort may be similarly challenging due to factors such as confusion, dementia or delirium.

Our categorisation of data including residential status and mobility status may have also introduced some misclassification around residential and mobility outcomes. Use of novel measurements at admission can identify patients at risk of adverse outcomes, facilitating improved care planning. However, the identification of a positive outcome can be challenging based solely on residential or mobility status. The categories used in our study were not clearly ordinal, and it is possible that patient outcomes were erroneously miscategorised.

## Conclusions and Implications

Identification of probable sarcopenia by simple HGS testing is a useful additional predictor of post-operative outcomes, especially delirium. Inability to complete HGS assessment is associated with worse outcomes than low HGS. When combined with the NHFS, HGS may improve discrimination for post-operative outcomes, and further research is required to externally validate a combined, reweighted NHFS-HGS score. Identification of hip fracture patients who are sarcopenic at admission may allow for targeted interventions to improve peri-operative care and potentially improve post-operative outcomes.

Larger, more generalisable studies are needed, with collection of each component of the NHFS, enabling HGS to be incorporated into a modified NHFS score. Further studies would aim to collect residential, mobility and activity of daily living (ADL) data using recognised scoring systems such as Barthel index to give a more generalisable patient profile [26]. Previous studies have outlined the principles for resistance exercise training programmes deliverable to older adults with sarcopenia [27]. Exercise programme intervention has been shown to be feasible to deliver across numerous NHS physiotherapy services to community-dwelling older sarcopenic patients, generating actionable insights for service improvement [28]. Further studies could therefore include specific interventions designed to improve care in sarcopenic hip fracture patients and assess association with post-operative outcomes.

## Supplementary Information

Below is the link to the electronic supplementary material.

Supplementary file1 (DOCX 301 KB)

## Data Availability

No datasets were generated or analysed during the current study.
