# Supplementary material for: Sarcopenia status as assessed by hand grip strength enhances prediction of post-operative outcomes in hip fracture patients
Source: Aging Clin Exp Res. 2025 Dec 29;38(1):9. doi: 10.1007/s40520-025-03262-7 (PMC12748096; doi:10.1007/s40520-025-03262-7)
Supplement: Supplementary file 1 — Supplementary file1 (DOCX 301 KB) [file 40520_2025_3262_MOESM1_ESM.docx]

**Supplementary Material**

**Title: “Sarcopenia status enhances prediction of post-operative outcomes in hip fracture patients”**

**Supplementary Table 1: Comparison of demographics, fracture characteristics and operations performed between included and excluded cohorts in study**

|  | Included (n=282) | Excluded (n=1287) | p |
| --- | --- | --- | --- |
| *Demographics* | | | |
| Age (SD) | 83.2±9.2 | 82.0±8.9 | **0.041** |
| Sex, Female (%) | 200 (70.9) | 902 (70.1) | 0.78 |
| Fracture Characteristics | | | |
| Fracture Type (%) |  |  |  |
| Intracapsular-displaced | 168 (59.6) | 632 (49.1) | **<0.001** |
| Intracapsular – undisplaced | 3 (1.1) | 26 (2.0) |  |
| Trochanteric- Grade A1/A2 | 97 (34.4) | 357 (27.7) |  |
| Trochanteric- Grade A3 | 11 (3.9) | 44 (3.4) |  |
| Subtrochanteric | 3 (1.1) | 59 (4.6) |  |
| Peri-prosthetic | 0 (0) | 103 (7.9) |  |
| Femoral fracture | 0 (0) | 66 (5.1) |  |
| *Operation Performed* | | | |
| Arthroplasty- Hemiarthroplasty | 154 (54.6) | 568 (44.1) | **<0.001** |
| Arthroplasty- Total hip replacement | 12 (4.3) | 49 (3.8) |  |
| Internal fixtion intramedullary nail | 17 (6.0) | 153 (11.9) |  |
| Internal fixation sliding hip screw | 97 (34.4) | 343 (26.7) |  |
| Other | 2 (0.7) | 113 (8.8) |  |
| No operation | 0 | 61 (4.7) |  |

Supplementary Table 1: Comparison between included and excluded cohorts detailing demographics, fracture characteristics and operation performed. Results displayed as mean ± standard deviation for parametric variables or N (%) for categorical values, with p-values recorded. While statically significant differences were noted between cohorts in age, these differences were deemed not clinically significant. Significant differences in fracture and operation types between cohorts were appropriately addressed using excluding criteria

**Supplementary table 2: Comparison of baseline assessment, sarcopenia assessment and post-operative outcomes between included and excluded study cohorts**

|  | | Included (n=282) | Excluded (n=1287) | p |
| --- | --- | --- | --- | --- |
| *Baseline assessments* | | | | |
| Pre-operative AMTS (IQR) | | 7.0 (2.0-10.0) | 9.0 (3.0-10.0) | **<0.001** |
| CFS (SD) | | 4.4±1.4 | 4.3±1.4 | 0.40 |
| CFS (%) | 1 | 10 (3.7) | 22 (1.9) | 0.40 |
|  | 2 | 19 (7.0) | 74 (6.5) |  |
|  | 3 | 44 (16.1) | 219 (19.2) |  |
|  | 4 | 65 (23.8) | 336 (29.4) |  |
|  | 5 | 61 (22.3) | 233 (20.4) |  |
|  | 6 | 65 (23.8) | 226 (19.8) |  |
|  | 7 | 9 (3.3) | 27 (2.4) |  |
|  | 8 | 0 (0) | 3 (0.3) |  |
|  | 9 | 0 (0) | 2 (0.2) |  |
| ASA Grade | 1 | 5 (1.8) | 18 (1.4) | 0.38 |
|  | 2 | 49 (17.4) | 256 (20.0) |  |
|  | 3 | 167 (59.2) | 693 (54.1) |  |
|  | 4 | 61 (21.6) | 308 (24.0) |  |
|  | 5 | 0 (0) | 7 (0.5) |  |
| NHFS (SD) | | 5.3±1.7 | 5.1±3.0 | 0.28 |
| NHFS, males only (SD) | | 6.0±1.6 | 5.6±1.6 | **0.045** |
| NHFS, females only (SD) | | 5.1±1.7 | 5.0±3.4 | 0.60 |
| *Sarcopenia assessment* | | | | |
| HGS (kg), males only (SD) n=55 | | N=55  23.5±6.3 | N=1  32.0±N/A | 0.19 |
| HGS (kg), females only (SD) | | N=118  16.1±5. | N=1  18.0±N/A | 0.75 |
| *Post-operative outcomes* | | | | |
| Mortality at trust discharge (%) | | 16 (5.7) | 101 (7.8) | 0.14 |
| 30-day mortality | | 20 (7.1) | 109 (8.5) | 0.33 |
| 120-day mportality | | 51 (18.1) | 216 (16.8) | 0.37 |
| Median length of stay (days) (IQR) | | 15.0 (8.0-24.0) | 14.0 (8.0-22.0) | 0.23 |
| Prolonged length of stay (>15 days) (%) | | 134 (47.5) | 537 (43.2) | 0.19 |
| Median 4AT Score (IQR) | | 3.0 (0.0-4.0) | 0.0 (0.0-4.0) | **<0.001** |
| Possible delirium (4AT 4-12) | | 130 (46.1) | 374 (31.7) | **<0.001** |

Supplementary Table 2: Comparison between included and excluded cohorts detailing baseline assessments, sarcopenia assessment and postoperative outcomes Results displayed as Mean ±SD) for parametric variables, Median (IQR) for nonparametric variables, or N (%) for categorical variables, with p-values recorded

**Supplementary table 3: Sex-stratified comparison of hand grip strength assessment, baseline assessment and baseline characteristics**

|  | | Males HGS (n=55) | | | Females with HGS (n=118) | | |
| --- | --- | --- | --- | --- | --- | --- | --- |
|  |  | n | HGS (kg)  (SD) | p value  *(r value)* | n | MaxGS (kg)  (SD) | p value  *(r value)* |
| *Baseline characteristics* | | | | | | | |
| Age | | 55 | 23.5 ±6.3 | 0.24  *(-0.16)* | 118 | 16.1 ±5.8) | **<0.001**  ***(-0.50)*** |
| Pre-fracture residential status | Own home/ sheltered housing  Residential care | 51  4 | 23.8 ±6.5  20.0 ±2.8 | 0.26 | 111  7 | 16.6 ±5.7  9.6 ±2.7 | **0.002** |
|  | Nursing care | 0 | N/A |  | 0 | N/A |  |
| Pre fracture residential status | Own home/ sheltered housing  Any other or dead | 51  4 | 23.8 ±6.5  20.0 ±2.8 | 0.26 | 111  7 | 16.6 ±5.7  9.6 ±2.7) | **<0.001** |
| Pre fracture mobility | Mobile outdoors without aids | 21 | 27.4 ±6.6 | **<0.001** | 42 | 18.9 ±6.1 | **<0.001** |
|  | Mobile outdoors with aids | 12 | 22.5 ±6.2 |  | 38 | 16.3 ±5.1 |  |
|  | Some indoor mobility | 22 | 20.3 ±3.9 |  | 37 | 12.8 ±4.3 |  |
|  | No mobility | 0 | N/A |  | 1 | 16.0 (N/A) |  |
| Pre fracture mobility | Mobile outdoors without aids  Any other | 21  34 | 27.4 ±6.6  21.1±4.9 | **<0.001** | 42  76 | 18.9 ±6.1  14.6 ±5.0 | **<0.001** |
| *Baseline Assessment* | | | | | | | |
| Pre-op AMTS | | 54 | 23.5 ±6.3 | **0.011**  ***(0.35)**** | 118 | 16.1 ±5.8 | **<0.001**  ***(0.33)**** |
| CFS | | 54 | 23.5 ±6.3 | **0.026 *(-0.30)*** | 114 | 16.1 ±5.8 | **<0.001**  ***(-0.51)*** |
| CFS | 1-4 “Not frail | 36 | 24.7 ±7.0 | 0.23 | 86 | 17.6 ±5.7 | **<0.001** |
|  | 5-6 “Mild-Moderately frail” | 17 | 21.7 ±4.0 |  | 27 | 11.9 ±4.0) |  |
|  | 7-9 “Severely frail” | 1 | 20.0 (N/A) |  | 1 | 10.0 (N/A) |  |
| ASA grade | Grade 1 | 0 | N/A | 0.56 | 4 | 22.0 ±9.1 | **<0.001** |
|  | Grade 2 | 10 | 25.2 ±6.7 |  | 34 | 19.4 ±5.2 |  |
|  | Grade 3 | 37 | 23.4 ±6.6 |  | 68 | 14.9 ±5.1 |  |
|  | Grade 4 | 8 | 22.0 ±4.4 |  | 12 | 12.1 ±4.1 |  |
| NHFS |  | 55 | 23.5 ±6.3 | 0.13  *(-0.21)* | 118 | 16.1 ±5.8 | **<0.001**  ***(-0.32)*** |

Supplementary table 3: Results of univariate analyses between HGS, demographics and baseline assessments performed following admission, total n=173. Statistically significant differences in HGS recorded (kg) seen with baseline AMTS and CFS in both sexes, and additionally with ASA grade and NHFS in female patients. Grip strength values recorded as Mean (±SD), with p-values recorded. * Correlation (‘r’) for non-parametric variables recorded using Spearman’s rank. All other correlations for parametric variables recorded using Pearson’s correlation coefficient.

**Supplementary table 4: Univariate associations between hand grip strength (not categorised into sarcopenia status) and post-operative outcomes, performed by sex**

| Post-operative outcomes | | | **Male (n=55)** | | | | | **Female (n=118)** | | | | |
| --- | --- | --- | --- | --- | --- | --- | --- | --- | --- | --- | --- | --- |
|  |  |  | **n** | | **HGS (kg) (SD)** | | **p value**  ***(r value)*** | **n** | **HGS (kg) (SD)** | | **p value**  ***(r value)*** | |
| 4AT delirium assessment score | | | 55 | | 23.5 ±6.3 | | **<0.001** | 118 | 16.1 ±5.8 | | **<0.001** | |
|  |  |  |  |  |  |  | ***(-0.46)**** |  |  |  | ***(-0.37)**** | |
| 4AT delirium assessment score | | 0 | 30 | | 26.2 ±6.5 | | **0.002** | 67 | 18.1 ±5.8 | | **<0.001** | |
|  |  | 1-3 | 16 | | 20.3 ±4.2 | |  | 27 | 14.2 ±5.1 | |  |  |
|  |  | 4-12 | 9 | | 20.2 ±5.1 | |  | 24 | 13.0 ±4.3 | |  |  |
| 4AT delirium score categorised | | 0-3  4-12 | 46  9 | | 24.1 ±6.4  20.2 ±5.1 | | 0.074 | 94  24 | 17.0 ±5.8  13.0 ±4.3 | | **0.003** | |
| Length of stay (LOS) | | | 55 | | 23.5 ±6.3 | | **0.008**  ***(-0.35)**** | 118 | 16.1 ±5.8 | | **<0.001**  ***(-0.33)**** | |
| LOS | Short (0-15 days)  Prolonged (>15 days) | | 28  27 | 24.9 ±5.1  22.1 ±7.3 | | **0.044** | | 67  51 | 17.1 ±5.7  14.9 ±5.6 | **0.046** | |  |
| Mortality at trust discharge | Alive  Dead | | 52  3 | 23.7 ±6.4  19.3 ±2.3 | | 0.25 | | 118  0 | 16.1 ±5.8  N/A | N/A | |  |
| 30-day mortality | Alive  Dead | | 52  3 | 23.5 ±6.4  23.3 ±6.1 | | 0.97 | | 118  0 | 16.1 ±5.8  N/A | N/A | |  |
| 120-day mortality | Alive  Dead | | 47  8 | 23.9 ±6.6  21.0 ±4.3 | | 0.23 | | 110  8 | 16.5 ±5.7  10.9 ±3.4 | **0.007** | |  |
| Trust discharge destination | Own home/ sheltered housing  Other | | 36  1 | 24.6 ±6.8  24.0 (N/A) | | 0.38 | | 81  1 | 17.6 ±5.6  18.0 (N/A) | **<0.001** | |  |
|  | Rehabilitation unit | | 7 | 24.0 ±6.7 | |  |  | 11 | 15.2 ±6.3 |  |  |  |
|  | Residential care | | 7 | 19.6 ±3.5 | |  |  | 22 | 11.7 ±3.9 |  |  |  |
|  | Nursing care | | 0 | N/A | |  |  | 1 | 8.0 (N/A) |  |  |  |
|  | Acute hospital | | 1 | 20.0 (N/A) | |  |  | 2 | 16.0 ±5.7 |  |  |  |
|  | Dead | | 3 | 19.3 ±2.3 | |  |  | 0 | N/A |  |  |  |
| 30-day residential status | Own home/ sheltered housing  Other | | 25  0 | 25.2 ±6.5  N/A | | 0.40 | | 71  1 | 17.6 ±5.7  18.0 (N/A) | **<0.001** | |  |
|  | Rehabilitation unit | | 18 | 22.7 ±6.7 | |  |  | 25 | 15.6 ±5.5 |  |  |  |
|  | Residential care | | 5 | 19.8 ±4.6 | |  |  | 18 | 10.5 ±3.1 |  |  |  |
|  | Nursing care | | 0 | N/A | |  |  | 0 | N/A |  |  |  |
|  | Acute hospital | | 4 | 21.5 ±4.4 | |  |  | 3 | 18.7 ±2.3 |  |  |  |
|  | Dead | | 3 | 23.3 ±6.3 | |  |  | 0 | N/A |  |  |  |
|  | Return to different than admission or dead | | 28 | 22.1 ±6.1 | |  |  | 40 | 14.7 ±5.2 |  |  |  |
| 120-day residential status | Own home/ sheltered housing  Other | | 28  0 | 24.0 ±5.5  N/A | | 0.26 | | 71  0 | 17.3 ±5.9  N/A | **<0.001** | |  |
|  | Rehabilitation unit | | 2 | 23.0 ±4.2 | |  |  | 2 | 18.5 ±10.6 |  |  |  |
|  | Residential care | | 6 | 20.2 ±4.2 | |  |  | 16 | 12.4 ±4.6 |  |  |  |
|  | Nursing care | | 0 | N/A | |  |  | 0 | N/A |  |  |  |
|  | Acute hospital | | 0 | N/A | |  |  | 0 | N/A |  |  |  |
|  | Dead | | 8 | 21.0 ±4.3 | |  |  | 8 | 10.9 ±3.4 |  |  |  |
| 120-day mobility | Mobile outdoors without aids  Mobile outdoors with aids | | 3  9 | 24.0 ±3.5  25.8 ±4.2 | | 0.33 | | 12  25 | 20.5 ±6.6  19.7 ±6.1 | **<0.001** | |  |
|  | Some indoor mobility | | 22 | 22.6 ±5.9 | |  |  | 43 | 13.6 ±4.5 |  |  |  |
|  | No mobility | | 0 | N/A | |  |  | 4 | 13.5 ±3.8 |  |  |  |

Supplementary table 4: Results of univariate analyses between hand grip strength and post-operative outcomes by sex.. Statistically significant differences in HGS recorded (kg) seen with baseline AMTS and CFS in both sexes, and additionally with ASA grade and NHFS in female patients. Grip strength values recorded as Mean (±SD), with p-values recorded. * Correlation (‘r’) for non-parametric variables recorded using Spearman’s rank. All other correlations for parametric variables recorded using Pearson’s correlation coefficient. Presence of delirium considered likely if 4AT score ≥4

**Supplementary table 5: Univariate analysis between probable sarcopenia (low hand grip strength) and post-operative outcomes, stratified by sex**

| **Sex** | **Sarcopenic status** | **Mortality** | | | | **Residential status** | | | | **120-day mobility** | | **Prolonged length of stay (>15 days)** | | **Post-operative delerium** | |
| --- | --- | --- | --- | --- | --- | --- | --- | --- | --- | --- | --- | --- | --- | --- | --- |
|  |  | **30-day** | | **120-day** | | **30-day** | | **120-day** | |  |  |  |  |  |  |
|  |  | **OR [95% CI]** | **p** | **OR [95% CI]** | **p** | **OR [95% CI]** | **p** | **OR [95% CI]** | **p** | **OR [95% CI]** | **p** | **OR [95% CI]** | **p** | **OR [95% CI]** | **p** |
| All patients | No sarcopenia* | REF | | REF | | REF | | REF | | REF | | REF | | REF | |
|  | Probable sarcopenia | 1.51  [0.13-16.92] | 0.74 | 13.04  [1.68-101.10] | **0.014** | 2.17  [1.16-4.04] | **0.015** | 6.38  [2.47-16.48] | **<0.001** | 2.79 [0.89-8.75] | 0.078 | 2.04 [1.10-3.79] | **0.024** | 3.41 [1.39-8.37] | **0.007** |
|  | Could not complete | 13.49  [1.75-103.75] | **0.012** | 34.53  [4.61-258.69] | **<0.001** | 21.78  [99.45-50.20] | **<0.001** | 71.86  [25.11-205.71] | **<0.001** | 15.58 [1.93-126.10] | **0.010** | 1.95 [1.06-3.58] | **0.031** | 77.37 [28.96-206.72] | **<0.001** |
| Men | No sarcopenia* | REF | | REF | | REF | | REF | | REF | | REF | | REF | |
|  | Probable sarcopenia | 0.81  [0.068-9.63] | 0.87 | 3.28  [0.37-29.12] | 0.29 | 1.29  [0.40-4.16] | 0.67 | 9.71  [1.12-84.30] | **0.039** | 1.05 [0.085-13.00] | 0.97 | 2.85 [0.83-9.76] | 0.096 | 3.87 [0.44-33.85] | 0.22 |
|  | Could not complete | 4.29  [0.47-39.39] | 0.20 | 8.82  [1.01-77.26] | **0.049** | 8.00  [1.70-37.67] | **0.009** | 80.67  [7.50-868.19] | **<0.001** | 1.40 [0.078-25.14] | 0.82 | 2.37 [0.65-9.69] | 0.19 | 52.50 [5.71-482.55] | **<0.001** |
| Women ~ | No sarcopenia* | REF | | REF | | REF | | REF | | REF | | REF | | REF | |
|  | Sarcopenia | - | - | - | - | 2.41  [1.13-5.15] | **0.023** | 5.42  [1.84-15.99] | **0.002** | 3.55 [0.89-14.18] | 0.074 | 1.76 [0.84-3.68] | 0.13 | 3.71 [1.35-10.19] | **0.011** |
|  | Could not complete | - | - | - | - | 30.55  [11.18-83.48] | **<0.001** | 71.00  [21.76-231.67] | **<0.001** | - | - | 1.85 [0.93-3.68] | 0.080 | 109.78 [33.55-359.15] | **<0.001** |

Supplementary table 5: Results of univariate binary logistic regression analysis between sarcopenic status (classified by hand grip strength) and selected post-operative outcomes. ~There was insuffient mortality for women patients at 30- and 120- days for binary regression analysis to be performed. Insufficient women unable to complete grip strength assessment were mobile outdoors without aids for regression analysis to be be computed.

**Supplementary Figure 1: Flowchart of included patients with data availability**

**
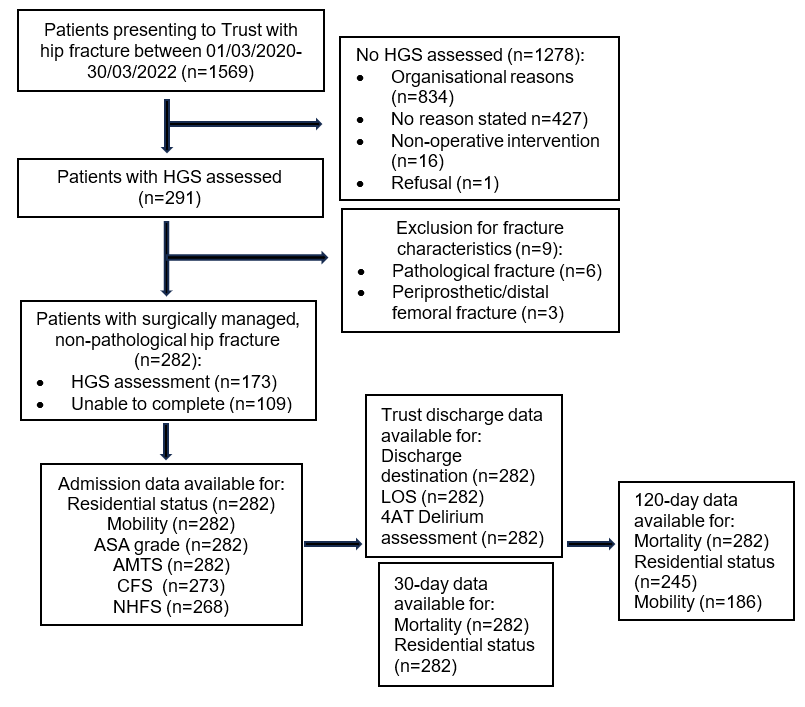
**

**Supplementary figure 1:** Number of patients included, with details of information available at each data collection point, and the number of patients excluded, with rationale provided. HGS: Hand grip strength ASA: American Society of Anaesthesiologists AMTS: abbreviated mental test score CFS: Clinical frailty score, NHFS: Nottingham hip fracture score, LOS:Length of stay

**Supplementary Figure 2: Visualised ROC Curve For 120-day mortality using the Nottingham hip fracture score alone, probable sarcopenia status as assessed by low hand grip strength and combined measure**


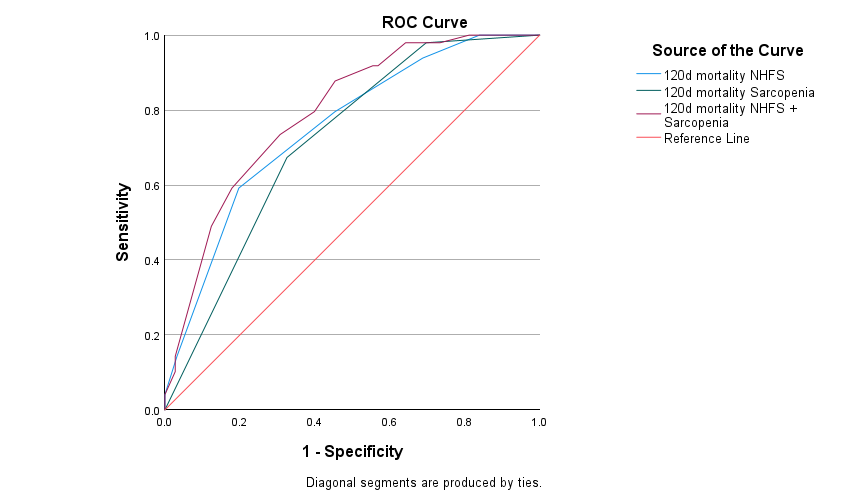


*Supplementary Figure 2: Curves for three prediction models for 120-day mortality, plotted against a reference line*

**Supplementary Figure 3: Visualised ROC Curve For 30-day residential status using the Nottingham hip fracture score alone, probable sarcopenia status as assessed by low hand grip strength and combined measure**


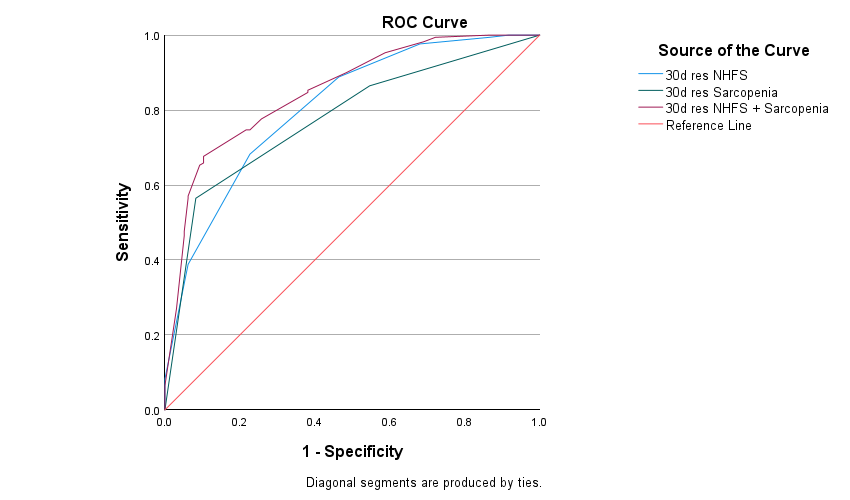


*Supplementary Figure 3: Curves for three prediction models for 30-day residential status, plotted against a reference line*

**Supplementary Figure 4: Visualised ROC Curve For 120-day residential status using the Nottingham hip fracture score alone, probable sarcopenia status as assessed by low hand grip strength and combined measure**


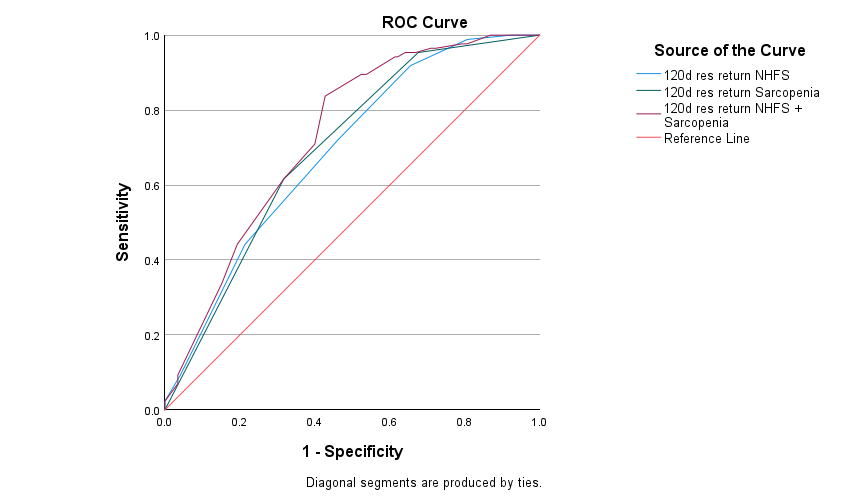


*Supplementary Figure 4: Curves for three prediction models for 120-day residential status, plotted against a reference line*

**Supplementary Figure 5: Visualised ROC Curve For 120-day mobility status using the Nottingham hip fracture score alone, probable sarcopenia status as assessed by low hand grip strength and combined measure**


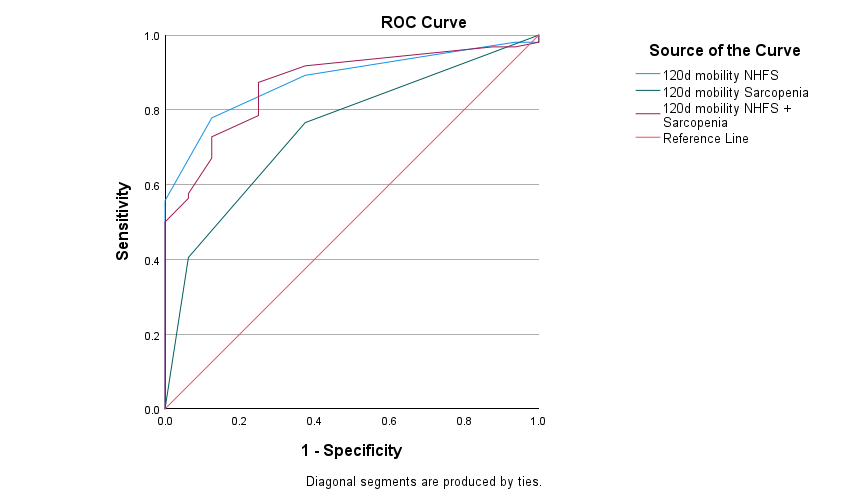


*Supplementary Figure 5: Curves for three prediction models for 120-day mobility, plotted against a reference line*

**Supplementary Figure 6: Visualised ROC Curve for prolonged length of stay (LOS) using the Nottingham hip fracture score alone, probable sarcopenia status as assessed by low hand grip strength and combined measure**


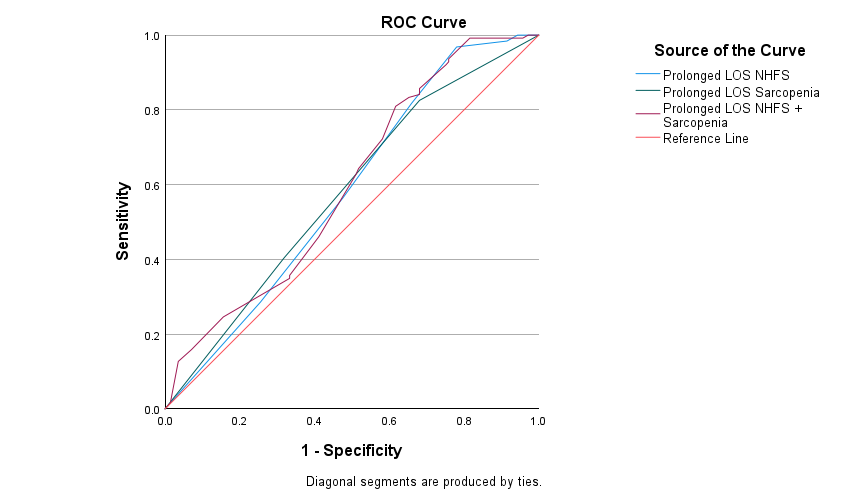


*Supplementary Figure 6: Curves for three prediction models for prolonged length of stay (Defined as >15 days), plotted against a reference line*

**Supplementary Figure 7: Visualised ROC Curve for post-operative delirium using the Nottingham hip fracture score alone, probable sarcopenia status as assessed by low hand grip strength and combined measure**


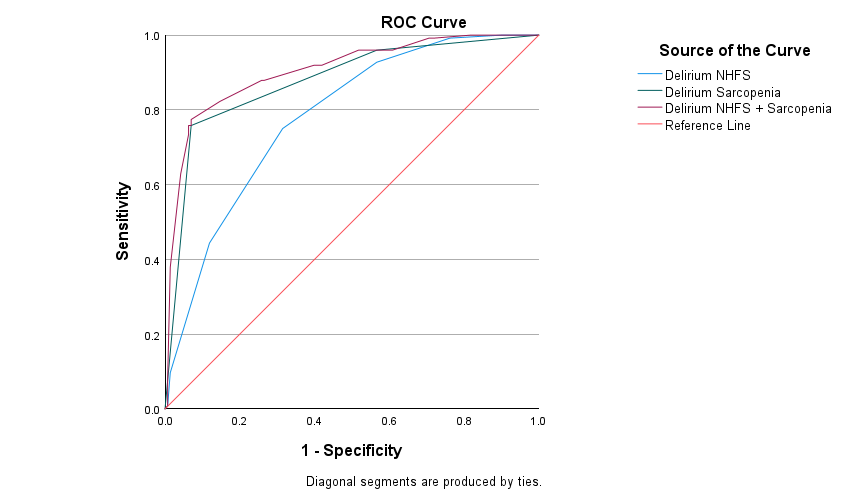


*Supplementary Figure 7: Curves for three prediction models for post-operative delirium (defined using 4AT score within 72h of surgery, with a 4AT≥4 indicating possible delirium), plotted against a reference line*
